# Chicago Medical College Commencement

**Published:** 1874-03

**Authors:** 


CHICAGO MEDICAL COLLEGE COMMENCEMENT. — The Commencement exercises of the Chicago Medical College (Med. Dept. of N.-W University) will take place on Monday and Tuesday, the 9th and 10th days of March, 1874. On Monday, the exercises will consist in the reading of theses, and the *public* examination of the candidates for graduation. The exercises on Tuesday will consist in the conferring of the degrees by the President of the University, a charge to the graduates, and a response by Mr. Kenny, as the representative of the class, and the formal valedictory address by Prof. W. P. Merriman. The exercises will commence at 2 o'clock, P. M., on both days, in the lecture room of the college. Members of the profession, and the community generally, are invited to attend.

